# Annual and Seasonal Trends in Mastitis Pathogens Isolated from Milk Samples from Dairy Cows of California’s San Joaquin Valley Dairies Between January 2009 and December 2023

**DOI:** 10.3390/vetsci12070609

**Published:** 2025-06-21

**Authors:** Daniela R. Bruno, Karen H. Tonooka, Terry W. Lehenbauer, Sharif S. Aly, Wagdy R. ElAshmawy

**Affiliations:** 1 Cooperative Extension, University of California Agriculture and Natural Resources, Fresno, CA 93710, USA; 2 Veterinary Medicine Teaching and Research Center, School of Veterinary Medicine, University of California Davis, Tulare, CA 93274, USA; khtonooka@ucdavis.edu (K.H.T.); tlehenbauer@ucdavis.edu (T.W.L.); saly@ucdavis.edu (S.S.A.); 3 Department of Population Health & Reproduction, School of Veterinary Medicine, University of California Davis, Davis, CA 95616, USA; 4 Department of Internal Medicine and Infectious Diseases, Faculty of Veterinary Medicine, Cairo University, Giza 12613, Egypt

**Keywords:** milk microbiology, dairy herd health, mastitis control

## Abstract

Bovine mastitis is a significant disease affecting dairy cattle worldwide, impacting milk quality and farm profitability. Understanding pathogen distribution is crucial for effective disease management. This study analyzed 319,634 individual cow milk samples submitted to the UC Davis Milk Quality Laboratory between 2009 and 2023 to assess pathogen prevalence, seasonal variations, and long-term trends. Routine microbiological cultures identified major and minor mastitis pathogens, with additional testing for *Mycoplasma* spp. Statistical analyses evaluated annual and seasonal trends in bacterial isolation rates. Results indicated that environmental pathogens, particularly non-aureus staphylococci and coliforms, were most frequently isolated, while contagious pathogens (*Staphylococcus aureus*, *Streptococcus agalactiae*, and *Mycoplasma* spp.) were less prevalent. Seasonal trends revealed higher contamination rates in Winter and increased no-growth samples in Summer. The study also observed a decline in sample submissions in recent years, possibly reflecting evolving dairy management practices. These findings provide a comprehensive perspective on mastitis pathogen dynamics in California’s Central Valley, supporting improved milk quality control measures and tailored mastitis prevention strategies.

## Simple Summary

This study examines the microbiological profiles of bovine milk samples submitted to the Milk Quality Laboratory at UC Davis between 2009 and 2023, analyzing over 319,000 samples. The research identifies long-term trends in mastitis-causing pathogens, seasonal variations, and shifts in contamination rates, providing crucial insights into dairy herd health. Findings reveal that environmental pathogens, particularly non-aureus staphylococci and coliforms, dominate mastitis cases, while contagious pathogens such as *Staphylococcus aureus*, *Streptococcus agalactiae*, and *Mycoplasma* spp. appear less frequently. Seasonal effects show higher contamination rates in Winter and increased no-growth samples in Summer, indicating environmental influences on pathogen prevalence. The study highlights a notable decline in sample submissions in recent years, possibly linked to evolving dairy practices and the rise in on-farm culturing techniques. These findings contribute valuable knowledge on pathogen dynamics, mastitis management, and milk quality control strategies in California’s Central Valley. By providing a 14-year perspective on bovine milk microbiology, the study supports dairy producers, veterinarians, and researchers in optimizing herd health and refining disease prevention approaches tailored to regional conditions.

## 1. Introduction

Mastitis is an economically significant disease affecting dairy cattle worldwide, impacting animal health, welfare, and productivity [1,2,3]. Bovine mastitis is most commonly caused by pathogens, typically classified as contagious or environmental based on their primary reservoir and transmission route [4]. Contagious mastitis pathogens reside in the cow’s udder and on teat skin, colonizing and growing in the teat canal. They are primarily transmitted among cows through contact with infected milk during milking [4]. Among these pathogens, *Staphylococcus aureus*, *Streptococcus agalactiae*, and *Mycoplasma bovis* are the most significant, with *S. aureus* considered to be the most common in North America [4,5]. On the other hand, environmental pathogens live in the cow’s environment, such as in the bedding and housing, and cause an infection when given the opportunity [4]. A wide range of bacterial species cause environmental mastitis, with the most common pathogens including coliforms (*Escherichia coli*, *Klebsiella* spp., *Enterobacter* spp.), *Streptococcus* species such as *Streptococcus uberis* and *Streptococcus dysgalactiae*, and *Pseudomonas* spp. [4]. Non-aureus staphylococci (NAS) are opportunistic bacteria that can cause intramammary infections [6]. Pathogens can also be categorized as major and minor based on their prevalence and the severity of the symptoms they cause [4].

Understanding the prevalence and distribution of mastitis-causing bacteria is crucial for controlling and preventing bovine mastitis [7]. Milk samples are often submitted for bacteriological examination to identify the causative agent as a part of mastitis control programs. However, limited information is available on the distribution of mastitis-causing organisms in milk from individual dairy cows in North America [8]. In California, one of the largest dairy-producing states, mastitis management is critical for maintaining productivity and profitability. California is the leading milk-producing state in the nation, housing 1.69 million dairy cows and accounting for 18% of the total milk produced in the United States. Over 90% of the cows are housed in dairies in the San Joaquin Valley [9]. Moreover, the California dairy industry has undergone significant structural transformations over the past two decades [10], including changes in environmental regulations and water usage, leading to shifts in bedding management and housing systems. Technological innovations, such as advances in milking technology and hygiene practices, alongside the adoption of genomic testing and improved diagnostic tools, have enhanced the ability to detect and manage mastitis. These advancements have led to better control of both environmental and contagious pathogens, likely influencing the health and management of dairy cows, as well as the profile of udder pathogens affecting them.

Therefore, the objective of this retrospective study was to describe the microbiologic culture results of dairy cows’ milk samples submitted from San Joaquin Valley dairies for routine microbiological testing to the Milk Quality Laboratory at the Veterinary Medicine Teaching and Research Center, UC Davis, Tulare, and to identify the prevalence, seasonal distribution, and annual trends of the most common mastitis pathogens isolated between January 2009 and December 2023.

## 2. Materials and Methods

The current retrospective study reviewed records of quarter milk samples submitted by dairy farms and veterinarians to the Milk Quality Laboratory at the Veterinary Medicine Teaching and Research Center, UC Davis. It examined data from samples submitted between January 2009 and December 2023, focusing on routine microbiological testing and *Mycoplasma* spp. detection. The data represented cows from dairy farms located in California’s San Joaquin Valley that submitted samples to the Milk Quality Laboratory at the Veterinary Medicine Teaching and Research Center, UC Davis. However, information on the cows’ clinical conditions, treatment histories, and specific dairy management practices was unavailable. Any samples submitted for research purposes were excluded. The researchers did not contribute to the collection or submission of the milk samples. The study was conducted on laboratory records, and therefore, approval from the Institutional Animal Care and Use Committee was unnecessary.

### 2.1. Microbiological Evaluation

Milk samples were cultured according to the National Mastitis Council guidelines [11]. Briefly, milk samples were plated on quarter plates of bovine blood agar [Biological Media Services, Davis, CA, USA] using sterile cotton swabs. Plates were incubated at 37 °C, read at 18–24 h, and then again at 48 h after culture. Results were classified as no bacterial growth (NG) or positive for bacterial growth with one colony type (pure culture), two different colony types (mixed infection), or more than two different colony types (contaminated samples). The growing bacteria were identified by colony morphology, catalase test (3% Hydrogen Peroxide), KOH reactions(3% solution – Potassium Hydroxide pellets P250-500 g - Fisher Scientific, Waltham, MA, USA), and Gram stain (BD BBL gram stain kit - Fisher Scientific, Waltham, MA). *Staphylococcus aureus* and *S. agalactiae* were confirmed using rabbit plasma coagulase (BD BBK Coagulase Plasma- Fisher Scientific, Waltham, MA) and CAMP tests (Biological Media Services, UC Davis, Davis, CA, USA), respectively. *Mycoplasma* spp. culture was also performed according to NMC guidelines with modifications. Briefly, the mycoplasma culture from milk samples was carried out by plating a cotton swab of milk on a mycoplasma agar plate (Myco-D, Biological Media Services, UC Davis, Davis, CA). Swabs used for streaking were placed into the enrichment mycoplasma broth (3 mL aliquot from Biological Media Services, UC Davis, Davis, CA). Plates were incubated at 37 °C with 4% CO_2_ for up to 7 days. Enrichment mycoplasma broths were incubated in an aerobic chamber for 48 h, streaked onto Myco-D plates, and then placed in the incubator under the same conditions mentioned above for mycoplasma growth for up to 7 days. Plates were read twice, on days 4 and 7 of the incubation. Results were recorded as NG if, after 7 days, no colonies were visualized, as contaminated if colonies other than *Mycoplasma* spp. were visualized, and as positive if they showed any number of small translucent domes with dense centers (fried egg appearance). Fluorescent Antibody Staining (Biological Media Services, UC Davis, Davis, CA) was performed on a subset of samples for *Mycoplasma* spp. identification.

### 2.2. Data Management and Statistical Analysis

Records of the milk culture results from the UC Davis-VMTRC Milk Quality Laboratory were compiled using a spreadsheet (Microsoft^®^ Excel^®^ for Microsoft 365 MSO, Version 2308, Redmond, WA, USA). Each entry in the dataset represented the milk culture results for each sample collected from individual cows. The study represents milk samples of mastitis cases in dairy cows housed in the San Joaquin Valley in California and submitted to the UC Davis-VMTRC Milk Quality Laboratory.

Culture results were categorized based on colony growth as follows: pure culture (a single colony type), mixed culture (two different colony types), contaminated (at least three different colony types), or NG (no bacterial growth). The frequencies of these culture outcomes—pure, mixed, contaminated, and NG—were calculated over various years (2009–2023) and seasons (Winter, Spring, Summer, Fall). The bacterial growth of the pure culture was further classified into environmental mastitis pathogens (Gram-positive, Gram-negative, Fungi, Algae, and Yeast) and contagious mastitis pathogens (*S. aureus*, *S. agalactiae*, and *Mycoplasma* spp.). Further classification of pure cultures into major and minor mastitis pathogens was conducted according to a previously published study [4]. Colony growth types were compared across years of isolation and seasons (Winter, Spring, Summer, Fall) using a univariable logistic regression model that adjusted for multiple comparisons. Specifically, pairwise comparisons using the Bonferroni adjustment were employed to compare the differences in colony growth type (pure culture, mixed infections, contaminated, NG), Gram stain (Gram-positive, Gram-negative), major versus minor mastitis pathogens, and contagious versus environmental mastitis pathogens across years and seasons.

All statistical analyses were conducted with Stata statistical software (Stata Corp. 2023, Release 18, College Station, TX, USA). Graphs were created using Microsoft Excel 2010 (Microsoft^®^ Excel^®^ for Microsoft 365 MSO, Version 2308, Redmond, WA, USA).

## 3. Results

A total of 319,634 milk culture records from dairy cows’ milk samples submitted for routine culture to the University of California Milk Quality Laboratory between January 2009 and December 2023 were evaluated. Of these, 299,152 were also cultured for *Mycoplasma* spp. The number of samples submitted by year significantly decreased over time, with 2022 and 2023 being the years with the fewest submissions (Table 1).

Overall, samples submitted for routine culture were categorized as NG (27.44%), contaminated (5.96%), or yielding pure (47.74%) and mixed (18.86%) culture results (Table 1). The proportion of samples with no bacterial growth decreased over time, with the highest proportion in 2012 (34.37%) and the lowest in 2021 (19.41%). The proportion of contaminated samples slightly increased over time, peaking in 2020 (8.67%) and reaching its lowest in 2012 and 2017 (4.28 and 4.24%). 

### Overall Pathogen Prevalence

Microorganisms isolated as pure cultures were categorized by Gram staining (Gram-positive, Gram-negative, or other), origin (contagious or environmental), and infection type (major or minor pathogen). The study found that environmental pathogens were the most frequently isolated in all years compared to contagious mastitis pathogens (Table 2).

Among environmental pathogens, Gram-positive bacteria were the most commonly isolated pathogens (75.37%), while Gram-negative bacteria and other groups (Fungi, Algae, and Yeast) were isolated from 18.24% and 1.63% of the samples, respectively (Table 2). Most isolates (64.63%) were classified as minor pathogens, and the prevalence of major pathogens decreased from 43.57% to 32.39% from 2009 to 2023 (Table 3).

*Streptococcus* spp. was the most commonly isolated genus (Table 4), with its prevalence increasing from 26.07% in 2009 to 44.52% in 2023, peaking in 2022 at 49.47%. Coliforms were the second most prevalent major pathogens, with their occurrence decreasing from 57.08% in 2009 to 41.35% in 2023, reaching the highest level in 2010 and the lowest in 2022 (Table 4).

Among major pathogens, *S. aureus* was the most commonly detected, identified as a pure isolate in 11.06% of samples. Its prevalence varied over the years, peaking in 2015 at 20.29% and reaching its lowest level in 2013 at 3.54%. A similar pattern was observed for *Mycoplasma* spp., which peaked in 2017 at 3.36% and was lowest in 2020, with an overall prevalence of less than 2.0%. Only 12% of samples underwent further Mycoplasma speciation, revealing *Mycoplasma bovis* as the most commonly isolated species at 43.52%. (Table 4).

Among Streptococcus species, *Streptococcus uberis* was identified in a small proportion of samples, with the highest prevalence occurring in 2015 (7.20%). Other *Streptococcus* species were isolated in a larger percentage of samples. *Streptococcus agalactiae* accounted for 2.60% of the *Streptococcus* species and had the highest prevalence in 2016 (8.04%). (Table 5).

NAS were the most frequently isolated among minor pathogens, accounting for 97.14% of cases. Their prevalence increased from 96.76% in 2009 to 99.67% in 2023, reaching the highest proportion recorded that year. Meanwhile, the prevalence of other minor pathogens declined over the same period (Table 6).

Seasonal trends significantly influenced results. The percentage of contaminated samples was higher in Winter (7.72%) and lower in Summer (4.12%), Spring (4.43%), and Fall (5.92%) (*p* < 0.05). Milk samples were more likely to be characterized as NG in Summer (30.33%) than in Spring (28.04%), Fall (28.63%), and Winter (25.78%) (*p* < 0.05). (Table 7).

The proportion of pathogens also varied by month and season. Contagious and environmental Gram-negative pathogens were more likely to be isolated in Winter and less likely to be isolated in Summer. In contrast, environmental Gram-positive pathogens were more frequently isolated in Summer and less so in Winter. Other microorganisms (Fungi, Algae, and Yeast) were more commonly detected in Summer but were less likely to be found in Fall (Table 8).

**Figure 1:**
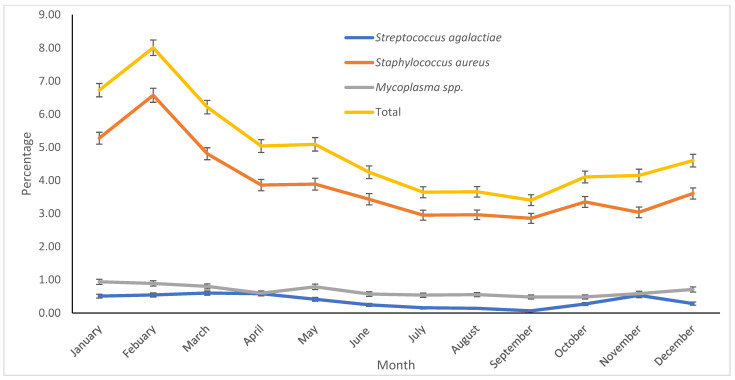
Monthly percentage of contagious mastitis pathogens (*Staphylococcus aureus*, *Streptococcus agalactiae*, and *Mycoplasma* spp.) from milk samples sent to the Milk Quality Laboratory at the Veterinary Medicine Teaching and Research Center, UC Davis, Tulare, California, between 2009 and 2023. Error bars represent one standard error.

*Streptococcus agalactiae* and *Staphylococcus aureus* were more frequently isolated in February, whereas *Mycoplasma* spp. showed minimal variation (Figure 1).

**Figure 2:**
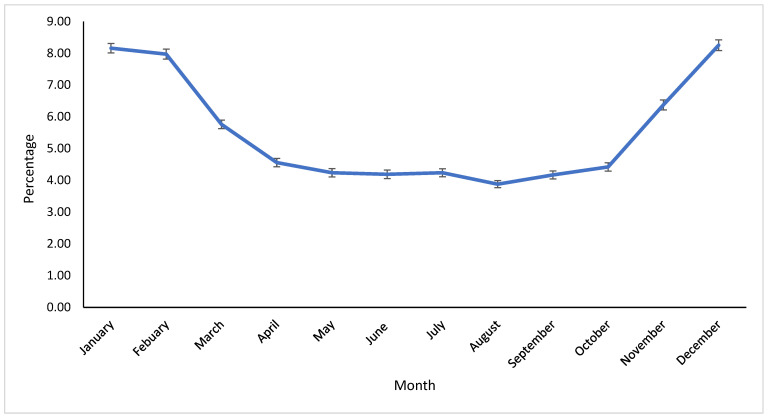
Monthly percentage of contaminated milk samples sent to the Milk Quality Laboratory of the Veterinary Medicine Teaching and Research Center, UC Davis, Tulare, California, from 2009 to 2023. Error bars indicate one standard error.

The highest percentage of contaminated samples occurred in the Winter months (December to January) (Figure 2).

## 4. Discussion

This is the first comprehensive study describing microbiological culture results from milk samples collected from dairy cows housed in California’s Central Valley. Understanding the profile of udder pathogens and monitoring trends over time is crucial for implementing effective mastitis control strategies. Milk samples are often collected from individual quarters or as composite milk samples (all quarters) to identify the pathogen causing an intramammary infection, or are taken from bulk tanks and string samples for herd surveillance. The current study evaluated records of milk samples from mastitis cases submitted to the Milk Quality Laboratory at the Veterinary Medicine Teaching and Research Center—the University of California, Davis, in Tulare, California, for routine milk culture between 2009 and 2023. The selection of the study laboratory was based on access to dairy cattle milk sample submissions. The study aimed to uncover patterns in the results of microbiological tests on milk samples originating from individual cows housed in California dairies. Notably, the results may underrepresent the prevalence of mastitis pathogens in California, as many dairy farms submit their milk samples to veterinary clinics or perform their own on-farm culturing.

Dairy farms rely on milk microbiological culturing to evaluate udder health status, which can be conducted using an on-farm culture (OFC) system or submitted to a diagnostic laboratory [12]. The popularity of performing an OFC has increased in recent years because it provides results within 24–48 h of sample collection. In contrast, laboratory-submitted samples may take several days from submission to the delivery of results to producers. The growing use of OFCs could partially explain the decline in the number of submissions from 2009 to 2023, which was similarly reported in a study evaluating trends of pathogen isolation in milk samples collected from cows in Canada [7]. Another factor that may have affected the number of submissions is a decrease in the number of dairies and animals in California over the years [9].

Conventional bacteriological culturing remains the gold standard for evaluating milk samples and identifying mastitis-causing pathogens. However, many samples yield no bacterial growth, complicating identification [13]. In this study, 27.44% of samples were NG, a result that is similar to findings from routine milk culture studies in Wisconsin [14] but lower than what was reported in a Canadian study [7]. A scoping review [15] analyzing MALDI-TOF results from 50,429 healthy cows and 43,924 clinical mastitis cases in Canada, the U.S., and Brazil found NG in 68.2% of healthy cow samples, but only 39.6% of clinical mastitis cases, with the latter aligning with our results. A recent California study on Gram-negative mastitis treatment in three large dairies reported NG rates ranging from 20.1% to 52.0% [2], suggesting variability based on farm conditions and sampling methods.

Several factors contribute to NG results in routine cultures, including infection clearance before sampling, prior antimicrobial use, improper sample handling, low bacterial loads, post-milking sampling, or cows not shedding the pathogen at the time of collection [13,14]. In this study, 98.68% of milk samples tested negative for *Mycoplasma* spp., consistent with a Cornell study [16] that found 98.03% of mastitic quarter samples showed no Mycoplasma growth. The low recovery of *Mycoplasma* spp. may be due to pathogen absence, intermittent shedding, or sample contamination, as Mycoplasma can be easily overgrown by other bacteria [17]. Seasonal trends also influenced NG results. Our study found a higher NG occurrence in Summer, aligning with a Canadian study [7], while a Wisconsin study [14] reported more NG cases in Winter. Regional climate differences may explain these variations—California’s Central Valley experiences hot, dry Summers exceeding 38 °C, whereas Wisconsin’s Summers are warm and wet (21–27 °C), and Canada’s range from 10 to 30 °C with high humidity.

Contamination of milk samples can occur at any point between collection and laboratory culturing. Common sources include the barn environment (e.g., feces, feed, bedding, air), teat skin, and the teat canal [18]. To minimize contamination, aseptic techniques should be followed during sample collection. Contaminated samples complicate pathogen identification, potentially delaying or misguiding treatment and masking the presence of major contagious pathogens. A recent study [15] reported contamination rates in U.S. dairies ranging from 2.5% to 39.7% for healthy cows and 2.4% for clinical mastitis cases. In our study, 5.63% of samples were contaminated, which was lower than in a similar Wisconsin study (15.3%) [14] but higher than findings from a Canadian study [7]. Contamination was more frequent in Winter, likely due to California’s wetter conditions. Since recycled manure is a common bedding material, increased organic matter on teats may contribute to higher contamination if teats are not properly cleaned. Interestingly, contamination rates were notably higher in 2020 and 2023. The COVID-19 pandemic in 2020 caused labor shortages and high turnover in the dairy industry [19,20], potentially impacting sample quality due to reduced training and workforce availability. Post-pandemic challenges continue to affect the agriculture sector [21], possibly influencing sample handling. A contaminated sample is typically identified when a culture yields three or more dissimilar colony types. Contamination can result from dirty teat ends, improper handling (e.g., milk touching hands before entering the tube), nonsterile equipment, contaminated media, excess alcohol on teat ends, and poorly sealed containers leading to alcohol evaporation. In this study, contamination remained below 10%, with most cases occurring in Winter, reinforcing the impact of seasonal conditions on sample quality.

Our findings showed that environmental pathogens were the most frequently isolated bacteria, which agrees with similar studies [7,14,15]. The environment plays a vital role in the growth and survival of environmental bacteria. Environmental mastitis pathogens are inherently present in the cows’ environment and often cause intramammary infections. Common environmental pathogens include major pathogens such as coliforms (*Escherichia coli*, *Klebsiella* spp., and *Enterobacter* spp.) and *Streptococcus* spp., as well as minor pathogens like NAS and *Corynebacterium* spp. The type of bedding, bedding management, and climate greatly influence the prevalence of specific bacterial populations. This significantly impacts udder health and mastitis incidence [22]. Organic materials such as composted manure bedding promote the rapid growth of environmental pathogens. Conversely, inorganic bedding, particularly sand, does not facilitate bacterial growth [23]. Composted manure bedding, also referred to as recycled manure bedding, is the most commonly used bedding in California dairies [24] and, if improperly managed, can be a substantial source of coliforms and other environmental pathogens.

Several Gram-negative pathogens can cause mastitis, with *Escherichia coli* and *Klebsiella* spp. being the most common, as they belong to a group commonly referred to as coliforms [4]. Many intramammary infections caused by Gram-negative bacteria develop into clinical mastitis [2]. Coliforms are the most common major environmental pathogens isolated in a pure culture. They naturally inhabit the soil and intestinal tract of animals, accumulating and multiplying in manure, as well as in contaminated bedding and water. *E. coli* is one of the leading causes of bovine mastitis and is found in the cow’s environment, including the bedding material, flies, alleys, and even the bovine gastrointestinal tract, which is a common reservoir for many environmental pathogens [25]. A recent study [4] evaluating the efficacy of intramammary therapy against Gram-negative bacteria reported that only 9.1% of the cases were attributed to Gram-negative bacteria, with over 90% of the isolates being *E. coli*. Wood-based bedding products are considered the primary source of *Klebsiella* spp. on dairy farms, although these bacteria can also be present in herds that use recycled manure or sand for bedding [26]. Any bedding contaminated with manure may contain *Klebsiella* spp., and the nutrients and moisture in bedding enhance the growth of coliforms. Research [26] has shown that healthy adult cows can shed Klebsiella organisms in their feces. The prevalence of *Klebsiella* spp. varies geographically due to differences in climate and management practices [27].

*Staphylococcus* spp. are differentiated in the lab into *S. aureus* and NAS for mastitis management. While *S. aureus* is considered a major contagious pathogen that causes a significant increase in somatic cell count and production losses, NAS are recognized as minor environmental mastitis pathogens, opportunists, and common skin inhabitants. They can be easily found in milking liners, the milker’s hands, bedding, floors, and air samples [6]. Although their importance in intramammary infections has not been clearly delineated, NAS have been associated with mild clinical and subclinical mastitis and a high elevation of quarter somatic cell count compared to uninfected quarters [6,28]. NAS were the most prevalent among all pathogens found in the present study, with an increase of 1.3-fold from 2009 to 2023. A Canadian study [7] reported a 17-fold increase in the prevalence of NAS from Canadian dairies between 2008 and 2017. A recent study also showed NAS as the most common pathogens isolated from clinical and subclinical cases of mastitis in Germany [29]. It appears that the prevalence of NAS has been increasing over the years; understanding the prevalence of this minor pathogen is therefore important for implementing prevention and control protocols.

*Streptococcus* spp. was the most frequently isolated Gram-positive species among major mastitis pathogens. It is commonly present on the mucosal surfaces and skin of animals and humans [30,31]. Specific tests are required to differentiate environmental *Streptococcus* spp., such as *S. uberis*, *S. dysgalactiae,* and other *Streptococcus* spp., from the contagious *S. agalactiae*. Several *Streptococci* species can cause bovine mastitis. However, there have been some instances where *S. uberis* acted as a contagious pathogen [30]. *Streptococcus uberis* and *S. agalactiae* can induce chronic mastitis [31].

The pathogens *Pseudomonas aeruginosa*, *Trueperella pyogenes*, *Nocardia* spp., *Mycobacterium*, *Serratia* spp., *Bacillus* spp., Fungi, Algae (*Prototheca* spp.), and Yeast are considered uncommon causes of mastitis, typically leading to sporadic infections that affect only a few cows within a herd. These opportunistic pathogens often exploit compromised udder health, such as teat injuries or suboptimal milking practices, to establish infections. Our study identified these pathogens in less than 2% of samples, indicating their relatively low significance as mastitis agents in the dairies. This observation aligns with findings from a survey of mastitis pathogens in Australia [32], which reported that the most prevalent isolates were *Streptococcus uberis*, *Staphylococcus aureus*, and *Escherichia coli*, with other pathogens constituting a minor fraction of cases. Additionally, *Prototheca zopfii* is recognized as an uncommon cause of bovine mastitis, typically leading to sporadic infections within herds. These findings underscore the importance of maintaining proper milking hygiene and udder health to prevent opportunistic infections by these less common mastitis pathogens.

The primary reservoir of contagious pathogens is the udders of infected cows, and transmission occurs during milking through the milkers’ hands, the liners of the milking unit, and cloths. This study found a low prevalence of contagious pathogens, which could be associated with effective mastitis control programs [33]. *Staphylococcus aureus*, a major mastitis pathogen, can cause a substantial economic loss once introduced into a herd [4,6]. It can colonize the scabs and damaged skin of cows, other animals, and humans and has been isolated from flies and environmental sites [34]. Moreover, *S. aureus* can be transmitted to heifers before calving by horn flies, and these infections can serve as a source of re-infection for cows in the herd [34]. Although it had low prevalence compared to environmental microorganisms, *S. aureus* was found to be the most common contagious mastitis pathogen isolated from cows in the study. We found a significant increase in the percentage of samples classified as positive for *S. aureus* from 2013 to 2015, decreasing thereafter. We suspect that some herds had outbreaks of this pathogen during this period.

*Streptococcus agalactiae* has been considered a very contagious obligatory intramammary pathogen primarily transmitted from cow to cow during milking, infecting many cows in the herd. However, recent reports suggest that it can be found in extramammary sources [30,31]. In our study, *S. agalactiae* was identified in a lower percentage (0.4%) of samples. *Streptococcus agalactiae* is often shed in high numbers in milk, leading to elevated bacterial counts in bulk tank milk [31].

*Mycoplasma bovis* and other *Mycoplasma* species have been reported as important contagious mastitis pathogens, with *M. bovis* being the most common species and likely causing the most severe mastitis problems [35]. *Mycoplasma* spp. have been detected in California dairies since the 1970s [35]. *Mycoplasma* spp. were also isolated in a small proportion of samples [1.02%], with the majority classified as *Mycoplasma bovis*. The low recovery of mycoplasma from the samples could be due to the methodology used (mycoplasma culture), as this method is relatively slow, often taking one to two weeks, with potential non-growth of these bacteria due to their fastidious culture requirements. However, mycoplasma mastitis should be suspected when milk samples from cows with clinical mastitis routinely test negative for pathogens by standard routine culture methods and when multiple quarters, often all four, are affected in individual cows. Other signs of Mycoplasma mastitis include sudden onset, rapid spread within the herd, a marked reduction in milk production, and resistance to treatment [15,16].

The incidence of clinical mastitis is greatly influenced by weather factors, which affect the seasonal isolation of mastitis-causing pathogens [36]. In our study, the season significantly impacted the proportion of pathogens. Most pathogens were isolated in Winter, except for NAS, which were primarily found in Summer. Reports suggest that the prevalence and distribution of pathogens vary greatly depending on the region [7,15,27]. Differences in the distribution of seasonal pathogens are likely associated with climatological variations. The Midwest and East Coast regions typically experience increased humidity and temperature in Summer, possibly leading to higher bacterial counts in bedding material. Conversely, Winter typically brings freezing conditions, reducing the bacterial population in the environment. California’s Winter tends to be humid, with precipitation levels that are higher than in other seasons, while Summer is usually dry and hot. Furthermore, recycled manure bedding on most dairy farms, combined with increased humidity during Winter, may elevate the risk of coliform exposure. It has been noted that the proportion of contagious pathogens worldwide has been decreasing, likely due to effective contagious mastitis control programs [33]. This suggests that the use of pre- and post-milking teat disinfectants, good milking hygiene, antimicrobial treatments, and dry cow therapy implemented in recent years could contribute to changes in the prevalence and distribution of contagious mastitis pathogens [37]. In our study, contagious pathogens (*S. aureus*, *S. agalactiae*, and *Mycoplasma* spp.), although isolated in low proportions, were more likely to be found in Winter. Infections acquired in Winter may persist into Spring, potentially increasing the likelihood of isolating contagious pathogens, as observed in the current study. The results of this study offer valuable insights into the prevalence and distribution of mastitis-causing pathogens in dairies within the Central Valley of California. The data indicate that both contagious and environmental pathogens are present, with significant variation in prevalence by season. These findings emphasize the importance of season-specific mastitis management strategies that address local pathogen profiles, particularly during Winter.

## 5. Conclusions

In conclusion, this study provides a comprehensive examination of the microbiological profiles of milk samples from dairy cows in California’s Central Valley over 14 years. The findings highlight the diverse range of pathogens responsible for mastitis in the region, with environmental pathogens being the most frequently isolated. While contagious pathogens were less prevalent, their presence underscores the importance of robust mastitis control programs. Seasonal variations in pathogen prevalence suggest that targeted, season-specific strategies are essential for managing udder health and effectively controlling mastitis. Overall, this study emphasizes the need for the continued monitoring of mastitis-causing pathogens to optimize prevention and treatment strategies tailored to the unique conditions of California dairies.

## Figures and Tables

**Table 1:** Annual percentage of no bacterial growth, pure cultures, mixed infections, and contaminated milk samples submitted to the Milk Quality Laboratory at the Veterinary Medicine Teaching and Research Center, UC Davis, Tulare, California.

Year	Routine Culture *	No Growth		Pure Culture		Mixed Culture		Contaminated	
		%	SE	%	SE	%	SE	%	SE
2009	33,907	26.68 ^ef^	0.24	48.04 ^ef^	0.27	18.63 ^d^	0.21	6.65 ^c^	0.14
2010	27,659	32.55 ^h^	0.28	46.83 ^bcde^	0.30	15.93 ^a^	0.22	4.69 ^ab^	0.13
2011	25,822	31.70 ^h^	0.29	46.96 ^cdef^	0.31	16.46 ^ab^	0.23	4.88 ^b^	0.13
2012	21,675	34.37 ^i^	0.32	45.77 ^abc^	0.34	15.58 ^a^	0.25	4.28 ^ab^	0.14
2013	14,729	27.49 ^f^	0.37	47.04 ^bcdef^	0.41	19.53 ^de^	0.33	5.94 ^c^	0.19
2014	17,326	33.20 ^hi^	0.36	46.18 ^abcd^	0.38	16.15 ^ab^	0.28	4.46 ^ab^	0.16
2015	19,404	29.53 ^g^	0.33	45.59 ^abc^	0.36	18.45 ^d^	0.28	6.43 ^c^	0.18
2016	22,559	26.50 ^ef^	0.29	47.78 ^def^	0.33	19.39 ^de^	0.26	6.33 ^c^	0.16
2017	34,342	30.07 ^g^	0.25	49.45 ^g^	0.27	16.24 ^ab^	0.20	4.24 ^a^	0.11
2018	27,795	27.35 ^f^	0.27	51.10 ^h^	0.30	17.14 ^bc^	0.23	4.41 ^ab^	0.12
2019	22,394	25.38 ^de^	0.29	48.43 ^fg^	0.33	20.19 ^e^	0.27	6.00 ^c^	0.16
2020	13,090	22.50 ^bc^	0.36	44.99 ^ab^	0.43	23.84 ^f^	0.37	8.67 ^d^	0.25
2021	20,622	19.41 ^a^	0.28	56.19 ^ah^	0.35	18.16 ^cd^	0.27	6.25 ^c^	0.17
2022	9225	20.89 ^ab^	0.42	47.61 ^cdefg^	0.52	23.49 ^f^	0.44	8.01 ^d^	0.28
2023	9085	24.02 ^cd^	0.45	44.15 ^a^	0.52	23.74 ^f^	0.45	8.09 ^d^	0.29
Total	319,634	27.44	0.30	47.74	0.40	18.86	0.30	5.96	0.20

* Total number of individual cow milk samples submitted to the laboratory for routine culture. Cells with the same letter within the same column mean that there is no significant difference, and cells with different letters mean that there is a significant difference.

**Table 2:** Annual percentage of environmental and contagious mastitis pathogens from milk samples sent to the Milk Quality Laboratory of the Veterinary Medicine Teaching and Research Center, UC Davis, Tulare, California, between 2009 and 2023.

Year	N *	Environmental Mastitis Pathogens												Contagious Mastitis Pathogens **			
		Gram-Positive				Gram-Negative				Fungi, Algae, and Yeast							
		%	SE	95%CI		%	SE	95%CI		%	SE	95%CI		%	SE	95%CI	
2009	16,288	65.39 ^e^	0.37	64.65	65.39	26.12 ^hi^	0.34	25.45	26.80	1.68 ^bc^	0.10	1.50	1.89	6.81 ^e^	0.20	6.43	7.21
2010	12,952	68.11 ^e^	0.41	67.30	68.11	27.04 ^i^	0.39	26.28	27.81	1.44 ^bc^	0.10	1.24	1.66	3.42 ^bc^	0.16	3.12	3.75
2011	12,126	71.30 ^a^	0.41	70.49	71.30	24.53 ^gh^	0.39	23.78	25.31	1.97 ^cd^	0.13	1.74	2.23	2.19 ^a^	0.13	1.95	2.47
2012	9920	72.56 ^ab^	0.45	71.67	72.56	23.78 ^g^	0.43	22.95	24.63	1.45 ^bc^	0.12	1.23	1.71	2.21 ^a^	0.15	1.94	2.52
2013	6929	71.79 ^a^	0.54	70.71	71.79	24.85 ^ghi^	0.52	23.85	25.88	1.17 ^b^	0.13	0.94	1.45	2.19 ^a^	0.18	1.87	2.57
2014	8002	76.18 ^c^	0.48	75.24	76.18	17.21 ^f^	0.42	16.40	18.05	1.51 ^bc^	0.14	1.27	1.80	5.10 ^d^	0.25	4.64	5.60
2015	8846	76.00 ^c^	0.45	75.10	76.00	14.88 ^cde^	0.38	14.15	15.63	1.45 ^bc^	0.13	1.22	1.72	7.68 ^ef^	0.28	7.14	8.25
2016	10,778	74.74 ^c^	0.42	73.91	74.74	14.98 ^cde^	0.34	14.32	15.67	2.75 ^ef^	0.16	2.45	3.07	7.53 ^ef^	0.25	7.05	8.05
2017	16,983	74.33 ^bc^	0.34	73.66	74.33	15.29 ^cde^	0.28	14.76	15.84	2.35 ^de^	0.12	2.13	2.59	8.03 ^f^	0.21	7.63	8.45
2018	14,204	74.98 ^c^	0.36	74.26	74.98	16.40 ^ef^	0.31	15.80	17.01	3.44 ^fg^	0.15	3.16	3.76	5.18 ^d^	0.19	4.83	5.56
2019	10,845	75.77 ^c^	0.41	74.95	75.77	16.27 ^def^	0.35	15.59	16.98	4.14 ^g^	0.19	3.78	4.53	3.82 ^bc^	0.18	3.47	4.19
2020	5889	82.65 ^d^	0.49	81.66	82.65	11.56 ^a^	0.42	10.77	12.41	0.44 ^a^	0.09	0.30	0.65	5.35 ^d^	0.29	4.80	5.95
2021	11,587	82.01 ^d^	0.36	81.30	82.01	14.59 ^c^	0.33	13.95	15.24	0.25 ^a^	0.05	0.17	0.36	3.15 ^b^	0.16	2.85	3.48
2022	4392	83.17 ^d^	0.56	82.04	83.17	12.02 ^ab^	0.49	11.09	13.02	0.23 ^a^	0.07	0.12	0.42	4.58 ^cd^	0.32	4.00	5.24
2023	4011	81.60 ^d^	0.61	80.37	81.60	14.09 ^bcd^	0.55	13.04	15.20	0.17 ^a^	0.07	0.08	0.37	4.14 ^bcd^	0.31	3.56	4.80

* N: Total number of samples with positive growth (pure colonies only). ** Contagious mastitis pathogens: only *Staphylococcus aureus*, *Streptococcus agalactiae*, and *Mycoplasma* spp. Cells with the same letter within the same column mean that there is no significant difference, and cells with different letters mean that there is a significant difference.

**Table 3:** Annual percentage of major and minor mastitis pathogens from milk samples sent to the Milk Quality Laboratory of the Veterinary Medicine Teaching and Research Center at UC Davis, Tulare, California, between 2009 and 2023.

Year	N *	Major				Minor			
		%	SE	95% CI		%	SE	95% CI	
2009	16,288	43.57 ^h^	0.39	42.81	44.33	56.43 ^a^	0.39	55.67	57.19
2010	12,952	43.20 ^gh^	0.44	42.35	44.05	56.80 ^ab^	0.44	55.95	57.65
2011	12,126	40.92 ^f^	0.45	40.05	41.80	59.08 ^c^	0.45	58.20	59.95
2012	9920	38.43 ^de^	0.49	37.47	39.39	61.57 ^de^	0.49	60.61	62.53
2013	6929	41.02 ^efg^	0.59	39.86	42.18	58.98 ^bcd^	0.59	57.82	60.14
2014	8002	32.17 ^abc^	0.52	31.15	33.20	67.83 ^fgh^	0.52	66.80	68.85
2015	8846	34.03 ^bc^	0.50	33.05	35.02	65.97 ^fg^	0.50	64.98	66.95
2016	10,778	34.32 ^c^	0.46	33.43	35.22	65.68 ^f^	0.46	64.78	66.57
2017	16,983	36.64 ^d^	0.37	35.92	37.36	63.36 ^e^	0.37	62.64	64.08
2018	14,204	33.68 ^bc^	0.40	32.91	34.46	66.32 ^fg^	0.40	65.54	67.09
2019	10,845	31.68 ^ab^	0.45	30.81	32.56	68.32 ^gh^	0.45	67.44	69.19
2020	5889	26.10 ^i^	0.57	24.99	27.24	73.90 ^i^	0.57	72.76	75.01
2021	11,587	30.48 ^a^	0.43	29.65	31.33	69.52 ^h^	0.43	68.67	70.35
2022	4392	31.81 ^abc^	0.70	30.45	33.20	68.19 ^fgh^	0.70	66.80	69.55
2023	4011	32.39 ^abc^	0.74	30.95	33.85	67.61 ^fgh^	0.74	66.15	69.05

* N: total number of samples with positive growth (pure colonies only). Cells with the same letter within the same column mean that there is no significant difference, and cells with different letters mean that there is a significant difference. Major pathogens: *Staphylococcus aureus*, *Streptococcus agalactiae*, *Streptococcus dysgalactiae*, *Mycoplasma bovis*, *Streptococcus uberis*, *Enterococcus* spp., *Proteus* spp., *Serratia* spp., *Yersinia* spp., *Pseudomonas aeruginosa*, *Truerperella pyogenes,* and Coliforms (*Escherichia coli*, *Klebsiella* spp., *Enterobacter* spp.). Minor pathogens: non-aureus staphylococci, *Corynebacterium* spp., Fungi, Algae, Yeast.

**Table 4:** Annual percentage of selected major mastitis pathogens from milk samples sent to the Milk Quality Laboratory at the Veterinary Medicine Teaching and Research Center, UC Davis, Tulare, California, between 2009 and 2023.

Year	N *	*Streptococcus* spp.				*Staphylococcus Aureus*				Coliforms				*Mycoplasma* spp.				Others			
		%	SE	95% CI		%	SE	95% CI		%	SE	95% CI		%	SE	95% CI		%	SE	95% CI	
2009	7216	26.07 ^h^	0.52	25.07	27.09	12.76 ^de^	0.39	12.01	13.55	57.08 ^f^	0.58	55.94	58.22	2.43 ^c^	0.18	2.09	2.81	1.66 ^bc^	0.15	1.39	1.99
2010	5670	31.22 ^a^	0.62	30.02	32.44	4.30 ^a^	0.27	3.81	4.86	60.37 ^g^	0.65	59.09	61.64	2.79 ^c^	0.22	2.39	3.25	1.32 ^abc^	0.15	1.06	1.66
2011	5020	35.52 ^bc^	0.68	34.21	36.85	3.63 ^a^	0.26	3.14	4.18	58.15 ^fg^	0.70	56.78	59.51	1.55 ^ab^	0.17	1.25	1.94	1.16 ^ab^	0.15	0.89	1.49
2012	3868	33.04 ^ab^	0.76	31.58	34.54	4.24 ^a^	0.32	3.65	4.92	60.13 ^fg^	0.79	58.58	61.67	1.14 ^ab^	0.17	0.85	1.53	1.45 ^abc^	0.19	1.12	1.88
2013	2879	35.19 ^abc^	0.89	33.46	36.95	3.54 ^a^	0.34	2.93	4.28	59.29 ^fg^	0.92	57.49	61.07	0.69 ^a^	0.15	0.45	1.07	1.29 ^abc^	0.21	0.93	1.77
2014	2607	32.83 ^ab^	0.92	31.06	34.66	14.58 ^def^	0.69	13.27	15.98	50.56 ^e^	0.98	48.64	52.47	0.77 ^a^	0.17	0.50	1.19	1.27 ^abc^	0.22	0.90	1.78
2015	3055	34.08 ^ab^	0.86	32.42	35.78	20.29 ^g^	0.73	18.91	21.76	42.26 ^bc^	0.89	40.52	44.02	1.90 ^cd^	0.25	1.47	2.45	1.47 ^aabc^	0.22	1.10	1.97
2016	3768	38.61 ^cde^	0.79	37.07	40.18	16.59 ^fg^	0.61	15.43	17.81	41.11 ^b^	0.80	39.55	42.69	1.86 ^de^	0.22	1.47	2.34	1.83 ^bc^	0.22	1.45	2.31
2017	6302	40.19 ^def^	0.62	38.99	41.41	15.49 ^f^	0.46	14.61	16.40	39.69 ^b^	0.62	38.48	40.90	3.36 ^ef^	0.23	2.95	3.84	1.27 ^abc^	0.14	1.02	1.58
2018	4891	39.01 ^de^	0.70	37.65	40.39	13.06 ^de^	0.48	12.15	14.04	44.71 ^cd^	0.71	43.33	46.11	1.02 ^a^	0.14	0.78	1.35	2.19 ^c^	0.21	1.81	2.64
2019	3510	38.95 ^cd^	0.82	37.35	40.57	10.11 ^bc^	0.51	9.16	11.16	47.49 ^de^	0.84	45.84	49.15	1.34 ^bc^	0.19	1.01	1.78	2.11 ^bc^	0.24	1.68	2.64
2020	1565	40.13 ^cdef^	1.24	37.73	42.58	16.23 ^efg^	0.93	14.48	18.14	41.66 ^bc^	1.25	39.24	44.12	0.19 ^a^	0.11	0.06	0.59	1.79 ^abc^	0.34	1.24	2.58
2021	3563	43.70 ^f^	0.83	42.08	45.33	7.80 ^b^	0.45	6.97	8.73	46.20 ^cde^	0.84	44.56	47.84	1.43 ^cd^	0.20	1.09	1.88	0.87 ^a^	0.16	0.61	1.23
2022	1413	49.47 ^g^	1.33	46.87	52.08	11.32 ^bcd^	0.84	9.77	13.08	35.39 ^a^	1.27	32.93	37.92	2.69 ^f^	0.43	1.96	3.67	1.13 ^abc^	0.28	0.69	1.84
2023	1323	44.52 ^efg^	1.37	41.86	47.21	11.94 ^cde^	0.89	10.30	13.80	41.35 ^abc^	1.35	38.72	44.02	0.38 ^abc^	0.17	0.16	0.90	1.81 ^abc^	0.37	1.22	2.69

* N: total number of samples submitted to the laboratory with major mastitis pathogens (pure colonies only). Cells with the same letter within the same column mean that there is no significant difference, and cells with different letters mean that there is a significant difference. Classification of major mastitis pathogens was carried out according to [4].

**Table 5:** Annual percentage of various *Streptococcus* spp. from milk samples sent to the Milk Quality Laboratory of the Veterinary Medicine Teaching and Research Center at UC Davis, Tulare, California, between 2009 and 2023.

Year	* N	*Streptococcus agalactiae*				*Streptococcus uberis*				Other *Streptococcus* Species			
		%	SE	95% CI		%	SE	95% CI		%	SE	95% CI	
2009	1881	0.69 ^a^	0.19	0.40	1.19	1.06 ^ab^	0.24	0.69	1.64	98.25 ^fg^	0.30	97.54	98.75
2010	1770	2.32 ^bc^	0.36	1.71	3.13	1.75 ^abc^	0.31	1.23	2.48	95.93 ^cde^	0.47	94.91	96.76
2011	1783	0.34 ^a^	0.14	0.15	0.75	0.95 ^ab^	0.23	0.59	1.53	98.71 ^g^	0.27	98.07	99.14
2012	1278	0.86 ^ab^	0.26	0.48	1.55	1.25 ^ab^	0.31	0.77	2.03	97.89 ^efg^	0.40	96.94	98.55
2013	1013	2.96 ^c^	0.53	2.08	4.20	0.89 ^ab^	0.29	0.46	1.70	96.15 ^cdef^	0.60	94.77	97.17
2014	856	0.93 ^abc^	0.33	0.47	1.86	0.47 ^a^	0.23	0.18	1.24	98.60 ^fg^	0.40	97.55	99.20
2015	1041	0.10 ^a^	0.10	0.01	0.68	7.20 ^f^	0.80	5.78	8.94	92.70 ^bc^	0.81	90.95	94.13
2016	1455	8.04 ^d^	0.71	6.75	9.55	3.99 ^ef^	0.51	3.09	5.12	87.97 ^a^	0.85	86.20	89.55
2017	2533	6.95 ^d^	0.51	6.02	8.01	3.43 ^de^	0.36	2.79	4.22	89.62 ^ab^	0.61	88.37	90.75
2018	1908	2.46 ^c^	0.35	1.86	3.26	1.94 ^bcde^	0.32	1.41	2.67	95.60 ^cd^	0.47	94.58	96.43
2019	1367	0.88 ^ab^	0.25	0.50	1.54	3.66 ^cde^	0.51	2.78	4.79	95.46 ^cd^	0.56	94.22	96.45
2020	628	9.24 ^d^	1.16	7.21	11.76	4.46 ^cdef^	0.82	3.10	6.38	86.31 ^a^	1.37	83.39	88.78
2021	1557	2.31 ^bc^	0.38	1.67	3.19	1.80 ^abcd^	0.34	1.24	2.59	95.89 ^cde^	0.50	94.78	96.77
2022	699	0.43 ^a^	0.25	0.14	1.32	2.72 ^abcde^	0.62	1.74	4.22	96.85 ^defg^	0.66	95.27	97.92
2023	589	0.51 ^a^	0.29	0.16	1.57	1.53 ^abcde^	0.51	0.80	2.91	97.96 ^defg^	0.58	96.45	98.84

* N: total number of samples submitted to the laboratory with *Streptococcus* spp. isolation (pure colonies only). Cells with the same letter within the same column mean that there is no significant difference, and cells with different letters mean that there is a significant difference.

**Table 6:** Annual percentage of selected minor mastitis pathogens from milk samples submitted to the Milk Quality Laboratory at the Veterinary Medicine Teaching and Research Center, UC Davis, Tulare, California, between 2009 and 2023.

Year	N *	Non-Aureus Staphylococci				*Corynebacterium* spp.				Fungi, Algae, Yeast			
		%	SE	95% CI		%	SE	95% CI		%	SE	95% CI	
2009	9072	96.76 ^cd^	0.19	96.37	97.10	0.23 ^abc^	0.05	0.15	0.35	3.01 ^cde^	0.18	2.68	3.38
2010	7282	97.25 ^de^	0.19	96.85	97.60	0.21 ^abc^	0.05	0.12	0.34	2.54 ^bcd^	0.18	2.20	2.93
2011	7106	96.34 ^cd^	0.22	95.88	96.75	0.30 ^abcd^	0.06	0.19	0.45	3.36 ^def^	0.21	2.97	3.81
2012	6052	97.22 ^ef^	0.21	96.78	97.61	0.41 ^bcde^	0.08	0.28	0.61	2.36 ^bcd^	0.20	2.01	2.78
2013	4050	97.80 ^ef^	0.23	97.30	98.21	0.20 ^abc^	0.07	0.10	0.39	2.00 ^b^	0.22	1.61	2.48
2014	5395	97.46 ^def^	0.21	97.01	97.85	0.30 ^abcd^	0.07	0.18	0.48	2.24 ^bc^	0.20	1.88	2.67
2015	5791	97.58 ^f^	0.20	97.15	97.95	0.21 ^abc^	0.06	0.12	0.36	2.21 ^bc^	0.19	1.86	2.62
2016	7010	95.45 ^bc^	0.25	94.94	95.91	0.36 ^abcd^	0.07	0.24	0.53	4.19 ^fg^	0.24	3.75	4.69
2017	10,681	95.62 ^cd^	0.20	95.21	95.99	0.65 ^de^	0.08	0.51	0.82	3.74 ^ef^	0.18	3.39	4.11
2018	9313	94.38 ^ab^	0.24	93.90	94.83	0.37 ^cd^	0.06	0.26	0.51	5.25 ^gh^	0.23	4.82	5.72
2019	7335	93.66 ^a^	0.28	93.08	94.20	0.22 ^abc^	0.05	0.13	0.36	6.12 ^h^	0.28	5.60	6.69
2020	4324	99.17 ^gh^	0.14	98.85	99.40	0.23 ^abc^	0.07	0.12	0.43	0.60 ^a^	0.12	0.41	0.88
2021	8024	98.83 ^g^	0.12	98.57	99.04	0.81 ^e^	0.10	0.64	1.03	0.36 ^a^	0.07	0.25	0.52
2022	2979	99.60 ^gh^	0.12	99.29	99.77	0.07 ^a^	0.05	0.02	0.27	0.34 ^a^	0.11	0.18	0.62
2023	2688	99.67 ^h^	0.11	99.36	99.83	0.07 ^ab^	0.05	0.02	0.30	0.26 ^a^	0.10	0.12	0.55

* N: total number of samples submitted to the laboratory with minor mastitis pathogens (pure colonies only). Cells with the same letter within the same column mean that there is no significant difference, and cells with different letters mean that there is a significant difference. Classification of minor mastitis pathogens was carried out according to [4].

**Table 7:** Seasonal percentages of pure cultures, mixed infections, no growth, and contaminated aerobic bacterial growth results from mastitis milk samples sent to the Milk Quality Laboratory of the Veterinary Medicine Teaching and Research Center, UC Davis, Tulare, California, between 2009 and 2023.

Season	N *	Pure Culture				Mixed Infection				No Growth				Contaminated			
		%	SE	95% CI		%	SE	95% CI		%	SE	95% CI		%	SE	95% CI	
Winter	89,454	45.93 ^a^	0.17	45.60	46.26	20.57 ^a^	0.14	20.30	20.83	25.78 ^a^	0.15	25.50	26.07	7.72 ^a^	0.09	7.55	7.90
Spring	75,084	50.32 ^b^	0.18	49.97	50.68	17.21 ^b^	0.14	16.94	17.48	28.04 ^b^	0.16	27.72	28.36	4.43 ^b^	0.08	4.28	4.58
Summer	79,117	49.36 ^c^	0.18	49.01	49.71	16.19 ^c^	0.13	15.94	16.45	30.33 ^c^	0.16	30.01	30.65	4.12 ^c^	0.07	3.98	4.26
Fall	75,979	47.16 ^d^	0.18	46.81	47.52	18.29 ^d^	0.14	18.02	18.57	28.63 ^b^	0.16	28.31	28.95	5.92 ^d^	0.09	5.76	6.09

* N: total number of samples submitted to the laboratory. Cells with the same letter within the same column mean that there is no significant difference, and cells with different letters mean that there is a significant difference.

**Table 8:** Seasonal percentages of contagious and environmental mastitis pathogens from milk samples sent to the Milk Quality Laboratory of the Veterinary Medicine Teaching and Research Center, UC Davis, Tulare, California, between 2009 and 2023.

Season	N *	Contagious Pathogens				Environmental Pathogens											
						Gram-Negative				Gram-Positive				Fungi Algae Yeast			
		%	SE	95% CI		%	SE	95% CI		%	SE	95% CI		%	SE	95% CI	
Winter	41,085	6.91 ^a^	0.13	6.67	7.16	20.71 ^b^	0.20	20.32	21.11	70.83 ^a^	0.22	70.39	71.27	1.55 ^a^	0.06	1.43	1.67
Spring	37,785	5.06 ^b^	0.11	4.84	5.29	19.43 ^a^	0.20	19.04	19.83	73.34 ^b^	0.23	72.89	73.78	2.17 ^b^	0.07	2.03	2.32
Summer	39,050	3.68 ^c^	0.10	3.50	3.87	16.70 ^c^	0.19	16.34	17.08	77.24 ^c^	0.21	76.83	77.66	2.37 ^b^	0.08	2.23	2.53
Fall	35,832	4.08 ^d^	0.10	3.88	4.29	19.26 ^a^	0.21	18.86	19.67	75.28 ^d^	0.23	74.83	75.72	1.38 ^a^	0.06	1.27	1.51

* N: total number of samples submitted to the laboratory (pure colonies only). Cells with the same letter within the same column mean that there is no significant difference, and cells with different letters mean that there is a significant difference.

## Data Availability

The data are available from the corresponding author.

## Abbreviations

The following abbreviations are used in this manuscript:

UC Davis	University of California Davis
VMTRC	Veterinary Medicine Teaching and Research Center
NMC	National Mastitis Council
NG	No growth
NAS	Non-aureus staphylococci
OFC	On-farm culture
SE	Standard Error
CI	Confidence Interval
USD	United States Dollar
MALDI-TOF	Matrix-Assisted Laser Desorption/Ionization Time-of-Flight
